# A combination of predispositions and exposures as responsible for acute eosinophilic pneumonia

**DOI:** 10.1186/2049-6958-9-7

**Published:** 2014-01-30

**Authors:** Simona Amiconi, Bertrand Hirl

**Affiliations:** 1 Department of Anesthesia and Critical Care, Schwabing Hospital, Munich 80804, Germany

**Keywords:** Acute eosinophilic pneumonia, Dendritic cells, Genetic predisposition, Pneumothorax

## Abstract

**Background:**

Acute eosinophilic pneumonia (AEP) is a rare febrile illness which is characterized by respiratory failure and often requires mechanical ventilation. The causes and sequence of events of this disease at a biochemical and histological level remain largely unknown. In this article we report the exceptional case, possibly unique, of a patient who developed AEP and three pneumothoraces within less than one month during her hospitalization.

**Case presentation:**

A 39-year-old German woman was admitted to our hospital for a laparoscopy-assisted vaginal hysterectomy under general anaesthesia. The surgical intervention was followed by peritonitis in the early postoperative course. Following anaesthesia induction with propofol/midazolam and during the prolonged therapy with several broad-spectrum antibiotics, she developed AEP and three spontaneous (one left-sided and two right-sided) pneumothoraces, the latter ones observed in quick succession. Symptoms, laboratory markers, and chest radiology significantly improved after a one-day treatment with methylprednisolone.

**Conclusions:**

On the whole, these pathological occurrences, together with similar cases reported in literature, can support the conclusion of possible predisposing genetic factors at the lung tissue level of AEP patients, a view that might shed new light on the pathogenesis of this disease. To provide a coherent pattern that explains the reported evidence for AEP and pneumothoraces, independently from the causative stimulus, the supposed molecular mutations could be localized in the connective tissue rather than in the epithelial cells. In order to interpret clinical and laboratory evidence, as well as to support the main conclusions, the important part of scientific research here presented can also assist physicians in making more informed decisions for the treatment of patients with pulmonary infiltrates.

## Background

Acute eosinophilic pneumonia (AEP) is a febrile illness leading to progressive, usually noninfectious respiratory failure that is characterized by diffuse pulmonary infiltrates with an increased number of eosinophils (> 25% of the total cell count) in the bronco-alveolar lavage (BAL) fluid, prompt response to corticosteroid administration, and absence of any relapse after recovery [1]. The AEP caused by drugs or toxins (drug-induced AEP) exhibits clinical, radiographic and histopathological features that are similar to the corresponding disease of unknown etiology (idiopathic AEP), often making the distinction between these two pathological entities difficult or ambiguous. The most common therapeutic substances that are suspected of causing drug-induced AEP are antibiotics and non-steroidal anti-inflammatory drugs [2]. Patients with idiopathic AEP frequently demonstrate recent changes in smoking habits (not only initiation of tobacco use, but also relapse after smoking cessation, increased daily smoking frequency or quantity, and short-term periods of passive smoking) [3].

Individuals who develop eosinophilic pneumonia may have underlying and undiscovered predispositions, because every day tens of thousands or even hundreds of thousands people are exposed to substances (medications or toxic products) or generic events (such as surgery, shock or sepsis) reported in association with AEP [4-6]. Altogether, only a small percentage of these exposed individuals ever becomes ill; indeed, AEP is an infrequent disease with only approximately 200 idiopathic cases reported worldwide since it was first described in 1989 [1]. This theory of the complex interaction between the genetic predisposition and the environmental (*e.g*., air-borne dust), external (*e.g*., drugs) or internal (*e.g*., biological mediators) stimuli has already been postulated for other lung disorders [7].

In line with this point of view, it has recently been described [8] the case of a Japanese girl of non-consanguineous family suffering from AEP and presenting loss-of-function mutations in a gene (*DOCK8*) located on chromosome 9. Other cases with eosinophilic lung diseases have also been observed in the absence of DOCK8 protein in lymphocytes [9]. Since patients with DOCK8 deficiency present defective T cell function, impaired production of antigen-specific antibodies and a severe defect in interstitial dendritic cell migration during immune responses with recurrent pulmonary infections, these reports suggest that AEP represents several pathophysiological processes that can fully and rightly be estimated only by understanding both the genetic and environmental contributions to its etiology and pathogenesis.

The eosinophil plays a key role (even though not fully understood) in the pathogenesis and clinical symptomatology of AEP. Blood eosinophilia is usually absent at the disease presentation [1], suggesting that the initial pathological stimulus originates at alveolar or pleural level. Both basic and applied research studies have demonstrated [10] that the selective activation of eosinophils is induced by several mediators, all of them sometimes categorized for simplicity with the most relevant of which, interleukin-5 (IL-5), *i.e*. with the name of the cytokine that exhibits the most specific and critical control of eosinophilic functions, including the regulation of eosinophilopoiesis in the bone marrow, the recruitment of these granulocytes to tissues, the lengthening of their survival by inhibiting apoptosis, and the differential activation and release of toxic products through their degranulation [11]. The local secretion of IL-5 and its enhanced concentration within restricted regions, detected in the broncho-alveolar lavage (BAL) fluid of patients with AEP, apparently reflects the presence of eosinophil-rich exudates in the alveoli andpleural cavity [12]. By contrast, IL-5 is usually undetectable in serum of patients who have AEP or eosinophilic pleural effusions, and IL-5 concentration decreases in BAL fluid after the resolution of symptoms [13]. These data suggest that the accumulation of eosinophils in the alveolar or pleural space is mainly governed by the local concentration of IL-5, the overexpression of which may be stimulated in the genetically hypersensitive tissue of predisposed subjects by an offending external/environmental factor [14].

We report the case of a non-smoking woman who, following a laparoscopy-assisted hysterectomy complicated by peritonitis, developed AEP and pneumothoraces after sedation with propofol and prolonged therapy with several broad-spectrum antibiotics. Observational evidence and plausible reasoning supporting a possible genetic predisposition to eosinophilic pneumonia is offered.

## Case presentation

**Figure 1:**
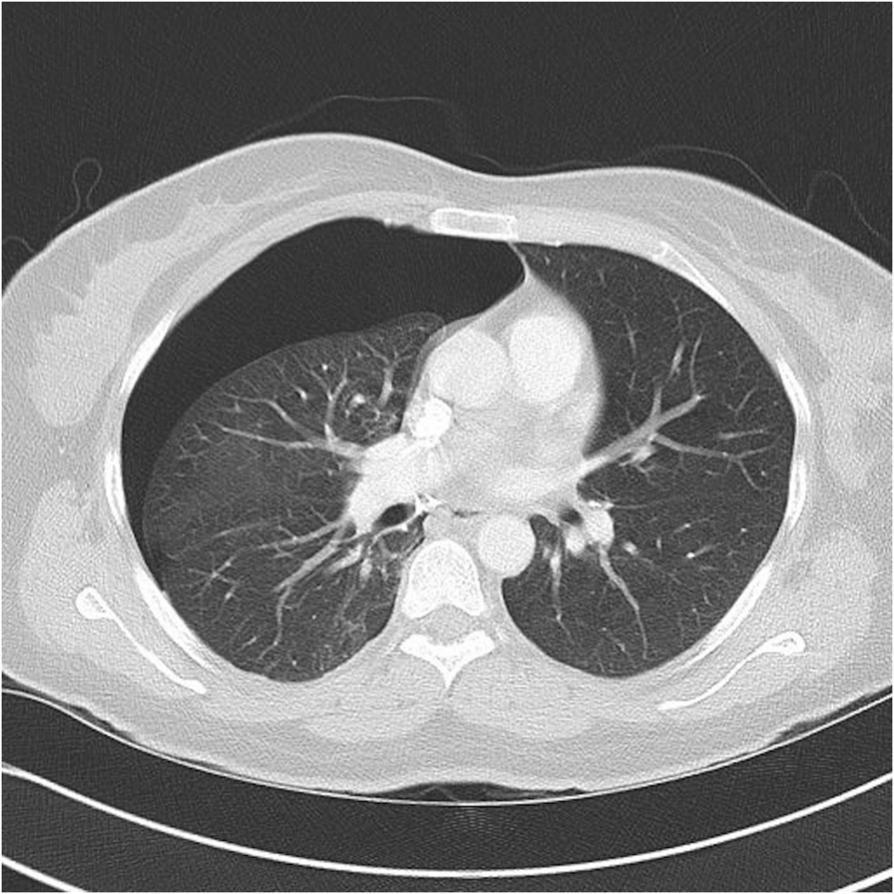
**High resolution computed tomography scan of the chest.** Large mantle pneumothorax at the level of the right middle lobe, observed 3 days after hysterectomy.

**Figure 2:**
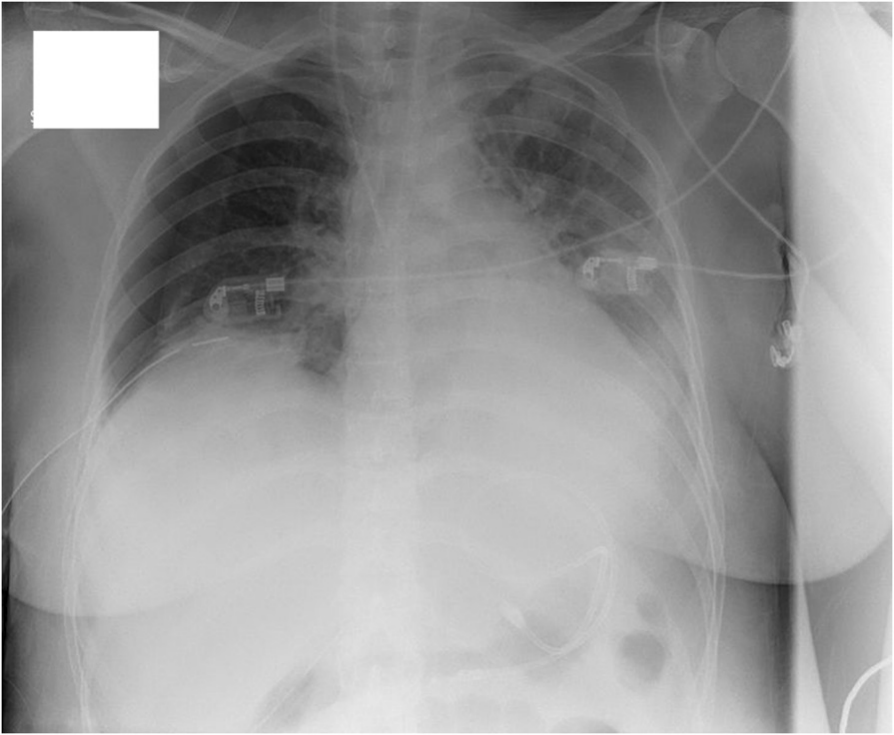
**Standard chest X**-**ray on the tenth postoperative day.** New mantle pneumothorax on the right side with diffuse interstitial infiltrates in mid field of the left lung and left pleural effusion. No cysts or bullae visible.

A 39-year-old woman was admitted to our hospital for a laparoscopy-assisted vaginal hysterectomy under general anaesthesia (induced with midazolam, fentanyl and propofol, and maintained with sevoflurane in oxygen/air mixture). The patient reported an unremarkable medical and family history and stated that she had never smoked. She also denied the following: (a) regular exposure to environmental tobacco smoke (at home or at her workplace), (b) abuse of illicit or recreational drugs, (c) food or medication allergies, (d) recent camping or wild animal contact. A more detailed history revealed that the patient was otherwise healthy and had taken no medications prior to the present illness. Three days after her surgery (with prophylactic ampicillin/sulbactam administered as *i.v*. bolus), the patient experienced acute epigastric pain with vomiting and diarrhoea along with dyspnea that gradually increased in severity. The patient had a temperature of 38.2°C, blood pressure of 90/60 mmHg, hearth rate of 120 beats/min, and respiratory frequency of 30 breaths/min. Inspection of her thorax revealed decreased movement of the chest wall, a hyperresonant percussion note, diminished fremitus and reduced breath sounds on the right side. The abdominal examination showed distension, guarding, and diffuse tenderness. The patient’s physical signs and radiographic findings were compatible with peritonitis and pneumothorax; the laboratory examination revealed a leukocyte count of 2,100 cells/μL, with 84% neutrophils and 3% eosinophils. A laparotomy revealed purulent fluid throughout the abdominal cavity, which was associated with fibrinous plaques covering most of the abdominal viscera; these findings were the result of a rectal perforation. A perforation closure was performed and peritoneal toilets (a 9-day-toilet plan) were initiated. The pneumothorax (Figure 1) was managed with a thoracostomy tube drainage. The patient was admitted to the intensive care unit (ICU) and began an empirical treatment with piperacillin and tazobactam. On the day of admission, an arterial blood analysis yielded PaO_2_ of 67 mmHg and SaO_2_ of 91.2% on ambient air. Non-invasive ventilation was started under moderate sedation with propofol. In the meantime, *Enterococcus* and *Escherichia coli* were cultured from the peritoneal exudate. On the seventh postoperative day, the patient’s clinical condition worsened, with increasing intra-abdominal fibrinous adhesions and paralytic ileus; the previous treatment was discontinued and an empirical administration of imipenem was begun on the conjecture of possible resistant enterococci. On the tenth postoperative day, she developed a new spontaneous pneumothorax on the right side (Figure 2), with respiratory rate increased to 28 breaths/min; the pathological event was managed with a thoracostomy tube drainage. Because the patient’s clinical condition were worsened, imipenem was discontinued on the twelfth day and substituted with tigecycline based on the antibiotic-sensitivity results for *Enterococcus faecium* isolated from the peritoneal toilet on the tenth postoperative day. On the eighteenth postoperative day, the patient’s respiratory distress increased (34 breaths/min and SaO_2_ of 88% with 6 L/min supplemental oxygen), her temperature was 37.6°C, her hearth rate 110 beats/min and her leukocyte count 19,400 cells/μL (neutrophils 86%; eosinophils 2%). Computed tomography (CT) of the chest revealed an abundant left-sided pleural effusion pressing on the lower pulmonary lobe, and multiple areas of alveolar infiltrates in the upper and middle right pulmonary lobes. The serology (hepatitis B virus, hepatitis C virus, human immunodeficiency virus, *Cytomegalovirus*, *Chlamydia* and *Mycoplasma*) eventually proved negative. Antinuclear antibody, anti-neutrophil cytoplasmatic antibody and rheumatoid factors were negative or non-specific. The levels of immunoglobulin [Ig] G, IgA, IgM, complement C3 and C4 were within the normal limits, and the IgE concentration was 112 kU/L. The CD4+/CD8+ T lymphocytes ratio was 1.7; since this value is usually significantly decreased in patients with various types of respiratory viral infections, the presence of influenza virus and/or bocavirus was not investigated.

**Figure 3:**
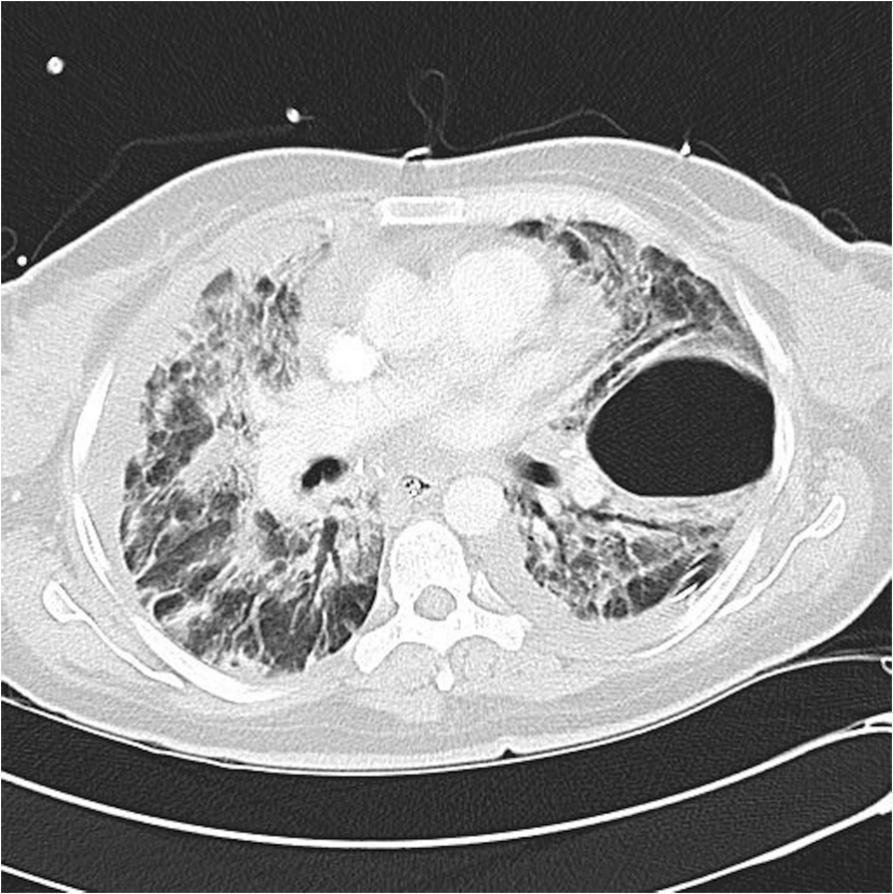
**High resolution computed tomography scan of the chest.** Extensive areas of air-space consolidations in both lungs and bilateral alveolar opacities. Pneumothorax at the level of the left lower lobe with bilateral pleural effusions.

**Figure 4:**
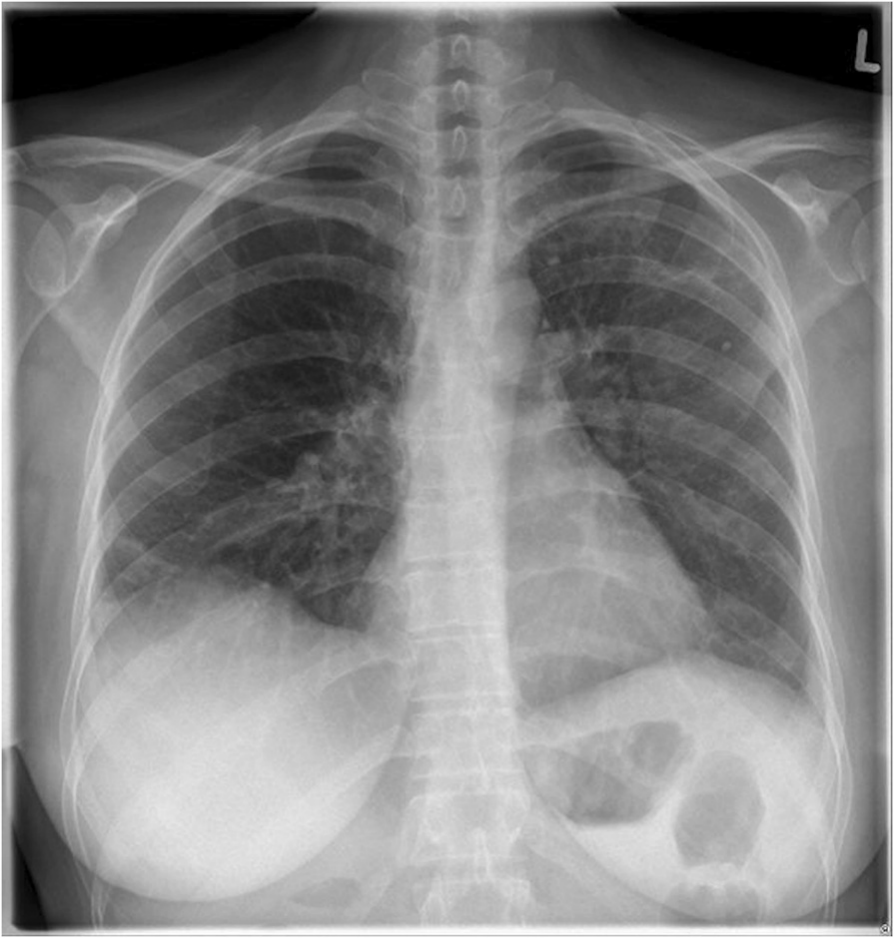
**Standard chest radiography performed on the day of discharge from the hospital.** Approximately one month after corticosteroid therapy, a dramatic improvement of pneumonic inflammation was observed with amelioration of bilateral infiltrates. Absence of pleural effusions.

The blood cultures for aerobic and anaerobic bacteria remained sterile. No pneumocystis was detected by polymerase chain reaction. The patient was then empirically treated withceftazidime and clarithromycin for atypical pneumonia, on the basis on the radiographic findings. Despite these measures, she did not improve and continued to have fever, with a temperature of approximately 37.5°C and mild leukocytosis. On the twenty-fifth postoperative day, a chest CT scan demonstrated a left-sided pneumothorax (Figure 3) with dense consolidations of the left upper and middle pulmonary lobes along with multiple areas of alveolar infiltrates in the upper and middle right pulmonary lobes. As the patient gradually became more dyspneic and hypoxic, she was placed under ventilation with continuous positive airway pressure. Instead of previous antibiotics, rifampicin and moxifloxacin were administered because of a positive test for *Legionella*, which, however, was refuted by laboratory on the following day. Because the patient’s respiratory failure further worsened, the previous treatment was discontinued and empirically substituted with three broad-spectrum antibiotics (linezolid, meropenem and moxifloxacin). Eosinophilic pneumonia was suspected when she showed no response to antibiotics within the next 2 days. Bronchoscopy demonstrated inflamed mucosa in both upper lungs with thick secretions in both lower lungs, and many nucleated cells (including 56% eosinophils) were detected in BAL fluid. These findings unequivocally supported a diagnosis of AEP, and parenteral methylprednisolone was initiated. Significant recovery occurred within the next 24 h, so the antibiotics were discontinued. The clinical symptoms further subsided and the patient was successfully extubated within 2 days after the initiation of steroids. The laboratory data and chest radiology revealed marked improvement. After 20 days in the surgery department, the patient was discharged (Figure 4) with a course of oral prednisone that was tapered for 14 days. At 1 year following surgery, the patient did not have any recurrence of her pulmonary symptoms or infiltrates.

## Discussion

An effective starting point for commenting our case might be the observation that our patient suffered from three pneumothoraces during her recovery in the ICU. Particularly, the first right-sided respiratory incident does not appear to have been secondary to carbon dioxide insufflation during the laparoscopy. Pneumothorax is a well-known, although infrequent, complication of abdominal laparoscopic surgery [15], with most pneumothoraces diagnosed postoperatively using conventional chest X-rays. In our case, the delayed onset of the pneumothorax on the third postoperative day suggests that this pathological event does not exhibit an etiopathogenesis due to carbon dioxide insufflation, but should probably be thought of as an adverse drug event associated with propofol and/or midazolam [16] administration for inducing the patient’s anaesthesia. Moreover, a characteristic clinical sign that could indicate the occurrence of pneumothorax during the laparoscopic surgery (*i.e*. alterations in the electrocardiographic patterns) was absent in the reported case; such a finding is a very sensitive indicator of intra-operative pneumothorax [15]. The risk of recurrence after the first spontaneous pneumothorax has been variably estimated [17] to be between 30% and 50% within the first 2 years; after one recurrence the risk of additional relapses within 3 years raises exponentially (to 62% after the first recurrence and to 82% after the second one). Although these intervals were not confirmed by other studies, the average time to recurrence after the first event has usually been spread over several years. The three pathological conditions, in which air accumulated within the thoracic cavity of our patient, thus appear unrelated to the general pattern. In our case, all three episodes occurred within three weeks, possibly due to etiologies, such as exposures to inciting factors and genetic predispositions, that should be examined in the patient herself and in the internal or external environments restricted to her recovery period in the ICU.

Doubts remain regarding the external factor that caused AEP in our patient. Several studies have emphasized the occurrence of drug-induced lung diseases [1]. Nonetheless, the clinician must have a high index of suspicion for medications in the etiology of AEP, including those drugs that have not been reported as causative agentsyet. Individual genetic deficiencies in drug-metabolizing enzymes may cause susceptibilities to certain pharmaceutical agents that require detoxification, therefore these drug toxicities may show unpredictable or idiosyncratic. In principle, all chemical agents taken during the days or weeks preceding the typical symptoms of AEP must be thoroughly investigated. AEP often develops within a few days of drug initiation. In theory, the only absolute proof that a drug is responsible for eosinophilic pneumonia may be obtained by linking its re-administration to ensuing relapses of the pneumonia, but this approach is unethical and even dangerous. The regression of eosinophilic pneumonia, after stopping the administration of a drug, is an indication of its iatrogenic cause. However, corticosteroids are often given concomitantly with drug withdrawals (as also occurred in our patient) to accelerate the clinical improvement, so the responsibility of a drug for AEP is rarely established definitively.

Numerous and diverse broad-spectrum antibiotics were administered for 23 days to our patient, because of peritonitis and progression of lung infiltrations (the latter showing no clinical improvement despite the pharmacological treatment). During her hospitalization in the ICU, she was treated with not fewer than four drugs, that have been reported to cause AEP or eosinophilia associated with parenchymal infiltrates: piperacillin/tazobactam [18], clarithromycin [19], moxifloxacin (a fluoroquinolone similar to levofloxacin [20]), and rifampicin [21]. In our patient many drugs were used simultaneously or in close sequence; thus, assigning toxicity to a specific agent was difficult. Therefore, the authors hesitate to indicate one or more of the various antibiotics as the cause of AEP and admit their inability to reach a final decision on this point.

Perhaps there was a possible genetic predispositions to the peculiar reactions of our patient’s pulmonary system. Although, to our patient’s knowledge, no previous episodes of pneumothorax had occurred in herself or in her relatives, the quick succession of all three episodes of pneumothorax - crowded within one month instead of years as for typical recurrences - strongly suggests a genetic predisposition to distinctive reactions of the pulmonary tissues, even though the underlying molecular defect remains only presumed. Moreover, genetic factors have been proposed as responsible for ordinary recurrences when located on the controlateral side [22]; in our case, a pattern of ipsilateral and controlateral respiratory incidents was observed. Primary spontaneous pneumothoraces (PSPs) may occur in patients with a variety of inherited disorders, such as metabolic disorders (*e.g*. individual abnormalities of the enzymes involved in biotransfomation) or connective tissue diseases [23]. Unfortunately, the latter could not be diagnosed because our patient was discharged before any consistent laboratory (*e.g*., electrophoretic analyses of skin biopsies that reveal a lack of type III collagen or elastin) or imaging (*e.g*., by electron microscopy) studies were performed. Therefore, only tentative proposals are in order. In our case, the precipitating cause for the first two pneumothoraces could be ascribed to drugs, *i.e*. propofol and/or midazolam [16], used for inducing anaesthesia (first pneumothorax) or for increasing the patient’s compliance during non-invasive ventilation (second pneumothorax). Presuming that the structural or functional proteins involved in the pathophysiology of pneumothorax carry mutations that make a potential contributory cause to the disease, attention should be focused on the genetic hyperreactivity of the pulmonary tissue rather than only on eosinophilicchemoattractants (*e.g*., high concentrations of IL-5) in the patient’s pleura or alveoli.

Additional anatomic considerations should be addressed. By definition, PSP is not associated with an underlying lung disease. However, this does not mean that there is no hidden pathological process. It has become increasingly apparent that PSP is associated with diffuse and often bilateral abnormalities within the pleura; in other words, it is not simply a disease caused by ruptured blebs/bullae, but includes inflammation and pleural porosity, *i.e*. areas of disrupted mesothelial cells in the visceral pleura that are replaced by an elastofibrotic layer with increased porosity, at level of which air leakage into the pleural space is allowed. The high resolution CT images of our patient’s chest, evaluated for the presence of possible parenchymal abnormalities (*e.g*., emphysematous bullae or cysts), were consistently negative. Therefore, in our patient the causes at histological level of PSPs are likely a combination of pleural porosities and inflammation. Moreover, pulmonary tissues appear related to one another, because they are derived from the endoderm and mesoderm, which are two of the three primary germ layers of the embryo. The endodermal lining of the lower respiratory system gives rise to the pulmonary surface epithelium, which in the terminal air sacs attenuates to an extremely thin squamous epithelial layer, thus forming the characteristic pulmonary alveoli. The lung connective tissue and the visceral and parietal pleura (including the blood and lymphatic vessels) are derived from the surrounding splanchnic mesenchyme (*i.e*., from the mesoderm). Thus, it is not surprising that the pathophysiology of PSP is coupled with inflammatory changes of the lungs and pleural cavity [24]. This structural/functional communication between the tissues of the pleura and lungs (based on the common embryonic origin of their connective tissue) is also expected to be reciprocal.

These latter remarks are confirmed and strengthened by comparing the pathological occurrences of our patient with cases of AEP/pneumothorax comorbidity, that are documented in the biomedical literature. A PubMed search, conducted using the search terms *acute eosinophilic pneumonia* and *pneumothorax*, resulted in 37 citations, but only in two studies [25,26] a true combination of these pathological events was established. In one case, a 55-year-old man [25] (i) developed the AEP symptoms three weeks after the beginning of treatment with the antidepressant trimipramine and (ii) suffered from a spontaneous pneumothorax fifteen days after this episode; when trimipramine was discontinued, the characteristic features of respiratory failure quickly subsided and the lung infiltrate disappeared. In the other case [26], a 14-month-old girl met the criteria for the diagnosis of idiophatic AEP, confirmed by lung biopsy, and of pneumothorax, verified by high-resolution computed tomography; in this case, such a disease cluster possibly occurred simultaneously. Areas of significant pulmonary abnormalities, such as blebs and bullae, were not described in these studies; therefore, in both cases the anatomic and histological state of pleural and lung tissues is comparable to that of our patient. In addition to this structural likeness, the pulmonary system of all patients taken here into account (*i.e*. that investigated in this work, as well as in [25] and in [26]) appears to share a common susceptibility to different agents, such as certain drugs and/or allergens, and to respond to these elements in a similar way, even though with different intensity (*e.g*., three pneumothoraces*versus* only one air leak). If it is how things stand, it follows that (i) PSP can precede [this work], or be simultaneous to [this work, and Ref. [26] or follows [25] AEP, and (ii) the attainment of comorbidity within one patient always occurs in a very short time (a few days or weeks, at latest). These data seem to imply that the pleura and lung form a functional *continuum* possibly through the connective tissue, within which elemental signals of inflammation migrate from a compartment (pleura or lung) to the other and *vice versa*.

On the whole, these considerations strongly suggest that all pathological events observed in our patient during her hospitalization (*i.e*., three pneumothoraces and AEP), reasonably reminiscent of analogous cases found in literature, are in line with the presence of a rare genetic predisposition at the molecular level of certain structures, possibly located into the connective tissue (see below) within her respiratory system.

A few conjectures address objections to the genetic influence on AEP. The following question arises: if some of the underlying etiologies of AEP are genetic, then why is the absence of recurrence so characteristic of this disease after corticosteroid treatment? Since the genetic features of the lung-tissue responsiveness can be suppressed but never destroyed, the abnormal reactivity is expected to recur whenever the inciting stimulus returns. However, several cases of AEP in which tobacco smoking was suspected to be the cause have shown no recurrence despite the reinitiation of smoking. These cases have been explained by the development of tolerance to tobacco [27] (however, without putting forward any sound mechanism), because the provocation tests (*i.e*., cigarette-smoking challenge tests designed to establish a causative link between AEP and the volatile constituents of cigarette smoke) were usually not associated with recurrent symptoms. By contrast, in drug-induced AEP, clinical improvement occurs with cessation of the offending agent, but symptoms usually recur after renewed administration of the medication. What then is the pathophysiological basis for interpreting these phenomenological differences between tobacco-induced and drug-stimulated AEP?

Alveolar and bronchial epithelial cells are injured through inhalation (*e.g*., by tobacco smoke or air-borne dust) or through the vascular system (*e.g*., by the medications). In AEP, the pathological changes at the epithelial level are potentially reversible. Once the inflammatory response has been suppressed by steroid treatment, an immediate tissue repair begins, involving the migration of the so-called type II epithelial cells adjacent to the area of damage (*i.e*., the stem cells of the alveolar epithelium) and leading to the complete restoration of the normal epithelial barrier function [28]*i.e*., the lung’s first line of defense against the external environment. In patients who have recovered from AEP caused by tobacco use, the resumption of smoking appears to elicit an unresponsive state [28], which may develop at the immune cells level in the lung parenchyma. Notably, specialized antigen-presenting cells (APCs), specifically the subset of dendritic cells (DCs), have emerged as central players in the immunological balance of the airways [29]. DCs include a heterogeneous family of professional APCs that are involved in the initiation of both immunity and immunological tolerance [30,31]. Compared with other cell types involved in the immune response, the tolerogenic DC (tDC) subset presents antigens that are captured in the “absence of microbial products or inflammatory mediators”*,* as typically occurs after the treatment with corticosteroids (*e.g*., in AEP cases) [32]. In particular, the resumption of smoking induces the following behavior at histological level: (i) migrating DCs interact with the resident alveolar cells and continually assess whether inhaled material should be confronted with an active immune response or whether homeostatic tolerance should be maintained (thus retaining an efficient low-alert surveillance mechanism that avoids an unnecessary inflammatory response), and (ii) the stimulation of survival signals from the alveolar epithelium locally enhances DCs survival.

As mentioned above, AEP is an adverse reaction only to abrupt modifications of smoking habits (particularly, the initiation of tobacco use or the increase in the daily smoking dose). In such cases, the alveolar cells are overwhelmed by copious amounts of tobacco-related toxic substances that cause vast injuries to the continuous single-cell layer of the epithelium (through the suppression of proliferation, apoptosis, or necrosis, depending on the concentration of volatile components from the cigarettes); consequently, extensive portions of the underlying mesodermally-derived connective tissue are exposed. According to this model, we hypothesize that this connective tissue is the target of the inciting factor that provokes AEP. Healthy individuals become susceptible to AEP during periods of marked variations in their smoking habits, because of the loss of alveolar epithelial cells (due to tobacco components) not sufficiently offset by the epithelial repair process; consequently, irritants from tobacco smoke can access extensive portions of the connective tissue. On the contrary, there is no physical barrier against the transport of drugs through the blood to the target tissue; therefore, the suspension or re-administration of an offending drug determines the improvement or relapse of the disease. Thus, the failure to relapse after a patient recovers from medication-induced AEP is strictly dependent on the complete cessation of any drug considered to be the most probable cause of the AEP episode; therefore, no tolerance to medications is expected.

## Conclusions

Our patient discloses the first observational collection of clinical data supporting the hypothesis that the medications used to treat overt disorders (*e.g*., peritonitis or lung infiltrations, as in our patient) or the exposure to tobacco smoke (but also fine, airborne sand or dust, as in the so-called idiopathic AEP) can all contribute to the development of non-infectious AEP, but “only in rare, genetically predisposed” subjects with mutations probably located within the connective tissue of the lung. An emerging consensus suggests that the described pattern of causative cofactors (*i.e*., the genetic predispositions or attributes and the environmental/external exposures to pathogenetic inducers) is common to certain lung diseases, such as asthma, chronic obstructive pulmonary disease and pulmonary fibrosis. We propose the addition of AEP to this list.

The genomic age is upon us. Therefore, if the mechanisms of the recruitment of inflammatory cells (such as eosinophils) in the airways are governed by the genetic characteristic of the pulmonary tissues, then genomic information in the diagnosis and treatment of AEP would seem nowadays necessary. In fact, physicians in some disciplines have already begun to use genetic profiles in their general practices, and in future many patients will most likely be analyzed for sequence information that outlines the genetic variations within their entire genomes. The promise of personalized medicine is a clinical reality. However, in our opinion, the use of genetic information to treat AEP patients would provide negligible profit because of the following factors: (i) the potential assistance in predicting the individual risk for AEP can fairly be disregarded because the prognosis is generally good; if however AEP is not considered in the differential diagnosis of respiratory failure, causing the disease to remain unrecognized with a consequent failure to administer corticosteroids to the patient, then death may occur, and (ii) full responsiveness to corticosteroids routinely occurs and relapses has never been described. Therefore, corticosteroids remain the mainstay of therapy for AEP, and there is no need for a personalized therapy.

## Consent

Written informed consent was obtained from the patient for publication of this case report and any accompanying images.

## Abbreviations

AEP: Acute eosinophilic pneumonia; APC: Antigen-presenting cell; BAL: Bronco-alveolar lavage; CT: Computed tomography; DC: Dendritic cell; IL-5: Interleukin-5; ICU: Intensive care unit; tDC: Tolerogenic dendritic cell.

## Competing interests

The authors declare that they have no competing interests. There has been no financial support to this work.

## Authors’ contributions

SA: study conception and design, analysis and interpretation of data, writing the manuscript; BH: critical revision of the paper. Both authors gave the final approval of the version to be published.
